# Psychotic symptoms in patient with nephronophthisis-related chronic renal disease

**DOI:** 10.1192/j.eurpsy.2026.11052

**Published:** 2026-07-31

**Authors:** B. Lázaro Alonso, J. Torres Cortés, I. Esteban Avendaño, E. Samper Pinilla

**Affiliations:** Hospital Universitario Ramón y Cajal, Madrid, Spain

## Abstract

**Introduction:**

We present the case of a 23 year-old woman with no previous psychiatric history admitted to nephrology to study the chronic renal disease she presents, related to untreated nephronophthisis. She abruptly begins with psychotic symptoms aproximately two weeks before admission, in the form of visual hallucinations and persecutory delusions. She also presents with involuntary movements and vague fears and worries. Speech is coherent at all times.

**Objectives:**

Liaison psychiatry is called upon during her stay for the differential diagnosis between primary or secondary psychotic symptoms in relation with CRD and a possible encephalopathy. During her hospitalisation she is evaluated by psychiatry, and low doses of antiphychotic are prescribed. On par with the stabilisation of renal function her psychotic symptoms also disappear, remaining completely asymptomatic at the time of this poster, even without antipsychotics.

**Methods:**

We analyse the existing literature regarding psychotic symptoms in relation with chronic renal disease, in search of possible associations between the two.

**Results:**

There is little literature regarding the association of chronic renal disease with psychiatric symptoms, and it is mostly focused on anxiety and sleep disturbances. We could not find any establishing a link between chronic renal disease and psychotic symptoms.

**Conclusions:**

We study the case of a young woman presenting with both chronic renal disease and psychotic symptoms. Both are treated during her hospital stay, achieving stabilisation or remission of symptoms. Despite searching the existing literature, we could not find a direct link between the two, pointing towards primary psychotic break.

**Disclosure of Interest:**

None Declared

